# Supplementary material for: A Novel Method for Creating a Synthetic L-DOPA Proteome and In Vitro Evidence of Incorporation
Source: Proteomes. 2021 May 24;9(2):24. doi: 10.3390/proteomes9020024 (PMC8162537; doi:10.3390/proteomes9020024)
Supplement: Supplementary file 1 [file proteomes-09-00024-s001.zip › Resubmission_Sup/Supplementry Table 1.docx]

Supplementry Table One A

Manually curated pathways of interest based on quantiative results from Mass Dynamics webserver. Project accessible at: <https://app.massdynamics.com/p/6f9b5fbf-ae94-4495-a0fe-0dd45cc55a07>.

| NAME | FDR | PROTEINS IN PATHWAY | FOUND IN LIST | VOLCANO PLOT |
| --- | --- | --- | --- | --- |
| [Metabolism of RNA](https://reactome.org/PathwayBrowser/#/R-HSA-8953854) | 7.90E-04 | 675 | 55 | 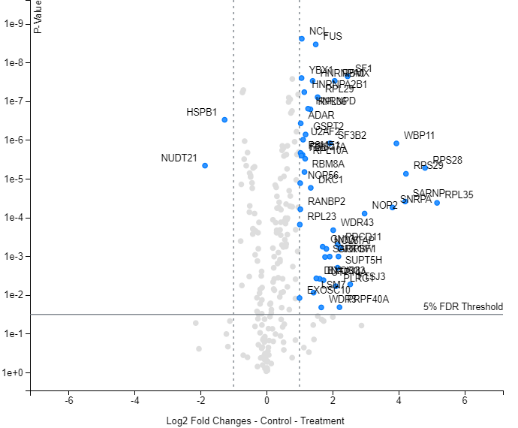 |
| [Processing of Capped Intron-Containing Pre-mRNA](https://reactome.org/PathwayBrowser/#/R-HSA-72203) | 0.006 | 245 | 26 | 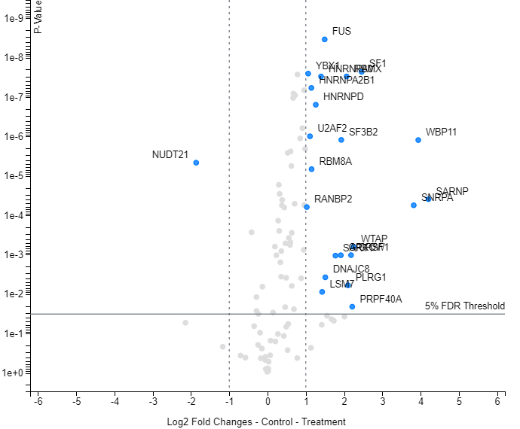 |
| [rRNA processing in the nucleus and cytosol](https://reactome.org/PathwayBrowser/#/R-HSA-8868773) | 0.006 | 193 | 22 | 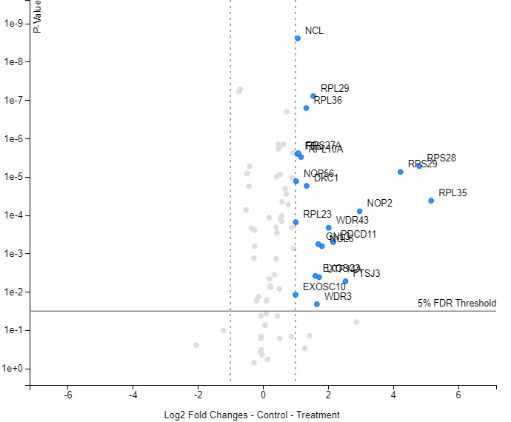 |
| [rRNA processing](https://reactome.org/PathwayBrowser/#/R-HSA-72312) | 0.01 | 203 | 22 | 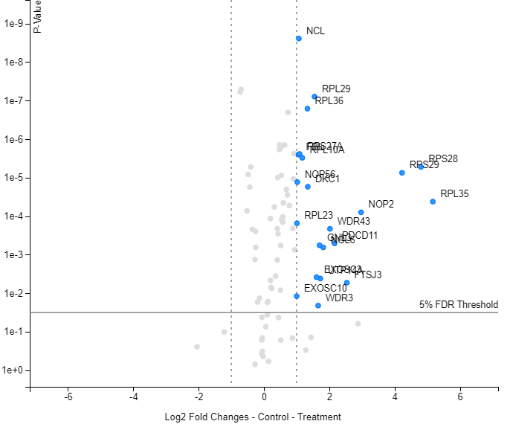 |
| [mRNA Splicing - Major Pathway](https://reactome.org/PathwayBrowser/#/R-HSA-72163) | 0.013 | 180 | 20 | 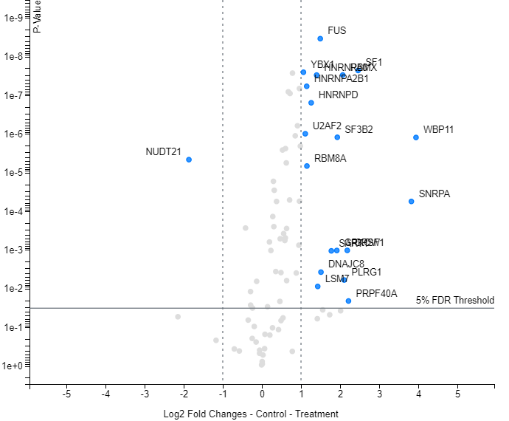 |
| [Major pathway of rRNA processing in the nucleolus and cytosol](https://reactome.org/PathwayBrowser/#/R-HSA-6791226) | 0.014 | 183 | 20 | 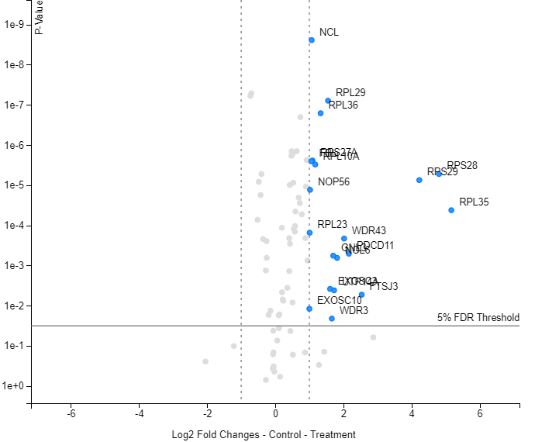 |
| [mRNA Splicing](https://reactome.org/PathwayBrowser/#/R-HSA-72172) | 0.017 | 188 | 20 | 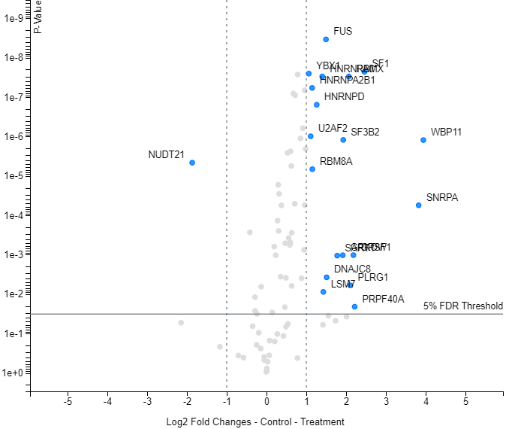 |
